# Manifestations of Racial Trauma in Bereaved Middle to Older Aged Black Adults

**DOI:** 10.1093/geroni/igab046.3157

**Published:** 2021-12-17

**Authors:** Danielle McDuffie

**Affiliations:** The University of Alabama, Tuscaloosa, Alabama, United States

## Abstract

Black adults have a higher likelihood of experiencing bereavement and increased negative implications of systemic racism compared to other groups. The effects of racism have also been suggested to have an impact on how bereaved Black individuals conceptualize their loss and the deceased. However, there is limited literature on how direct and indirect childhood experiences with racial violence and viewing racially violent deaths impact bereaved Black adults later in the lifespan. The current study seeks to explore the impacts childhood engagement with racial violence might have on bereaved middle to older Black adults. 103 middle to older aged Black adults (M=44.72, SD=5.48, 67% male) from a larger online grief study were probed about factors including somatization, depression, affect, grief, and the prevalence and intensity of exposure to race-based violence during their childhoods. Linear regressions and bivariate correlations were used for data analysis. Childhood racial violence significantly predicted grief (F=6.348, p=.013). Additionally, experiencing childhood racial violence was significantly associated with somatization (r=.197, p=.047), depression (r=.198, p=.045), and negative affect (r=.256, p=.010). Endorsed intensity of racial violence was significantly associated with depression and negative affect (r=.464, p=.000; r=.440, p=.000, respectively). Bereaved Black middle to older adults seem greatly impacted by childhood experiences of racial violence. It is important to consider the role outside cultural influences such as racial trauma might have on other deleterious mental health experiences such as bereavement. Furthermore, in the assessment of ACEs among Black and other people of color, it could be important to include childhood racial violence.

